# Supplementary figures and images for: The pH Effects on SARS-CoV and SARS-CoV-2 Spike Proteins in the Process of Binding to hACE2
Source: Res Sq. 2021 Sep 9:rs.3.rs-871118. Preprint. [Version 1] doi: 10.21203/rs.3.rs-871118/v1 (PMC8437318; doi:10.21203/rs.3.rs-871118/v1)

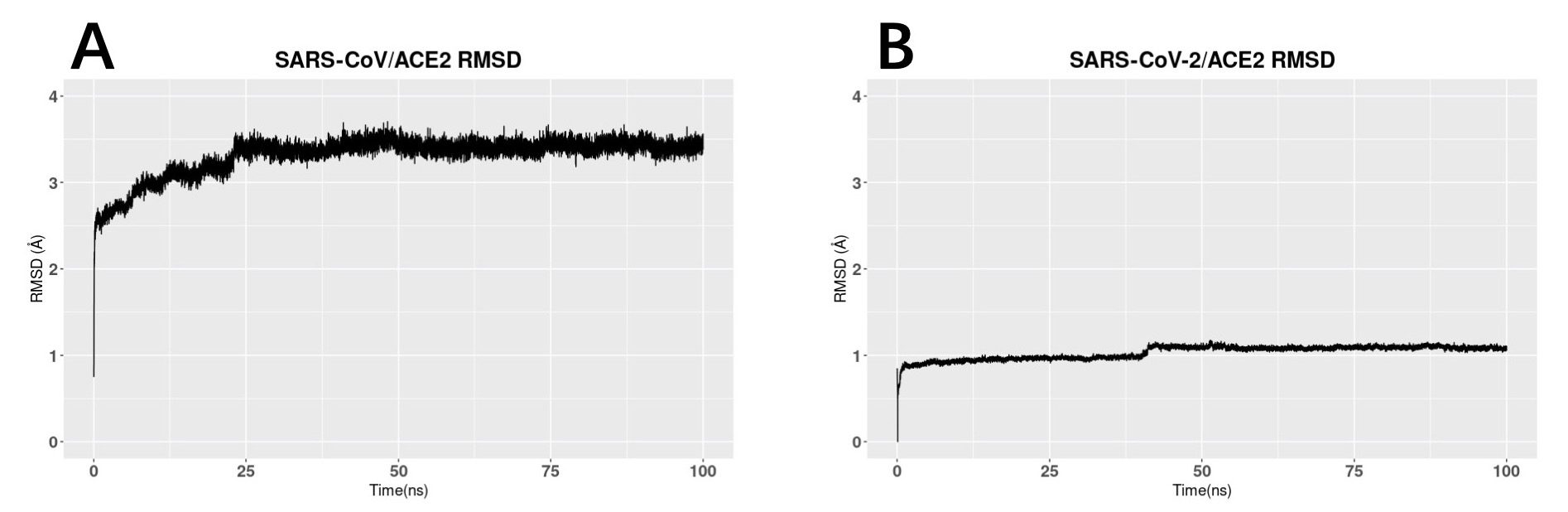

Supplement: Supplement 1 [file 7dd348e81feb3a861cec8dac.jpg]

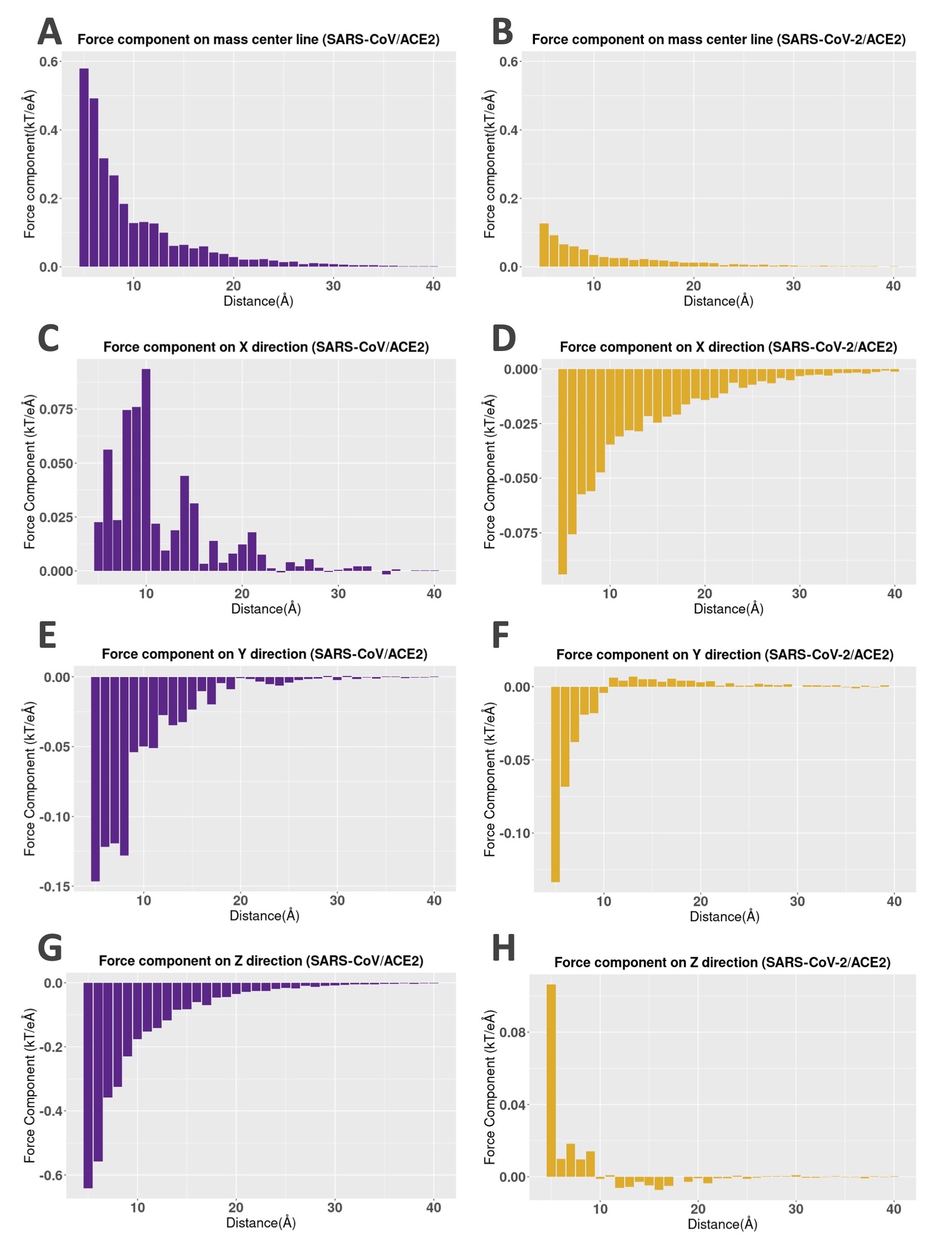

Supplement: Supplement 2 [file bb270869786d553c6081df59.jpg]
