# Supplementary material for: The pH Effects on SARS-CoV and SARS-CoV-2 Spike Proteins in the Process of Binding to hACE2
Source: Res Sq. 2021 Sep 9:rs.3.rs-871118. Preprint. [Version 1] doi: 10.21203/rs.3.rs-871118/v1 (PMC8437318; doi:10.21203/rs.3.rs-871118/v1)
Supplement: Supplement 3 [file 666b135112588bf700920cbb.docx]

**The pH Effects on SARS-CoV and SARS-CoV-2 Spike Proteins in the Process of Binding to hACE2**

**Yixin Xie^1^, Wenhan Guo^1^, Alan Lopez-Hernadez^1^, Shaolei Teng^2^, Lin Li^1,3*^**

^1^ Computational Science Program, University of Texas at El Paso, El Paso, TX.

^2^ Department of Biology, Howard University, Washington, D.C.

^3^ Department of Physics, University of Texas at El Paso, El Paso, TX.

***Correspondence:**

Lin Li: lli5@utep.edu


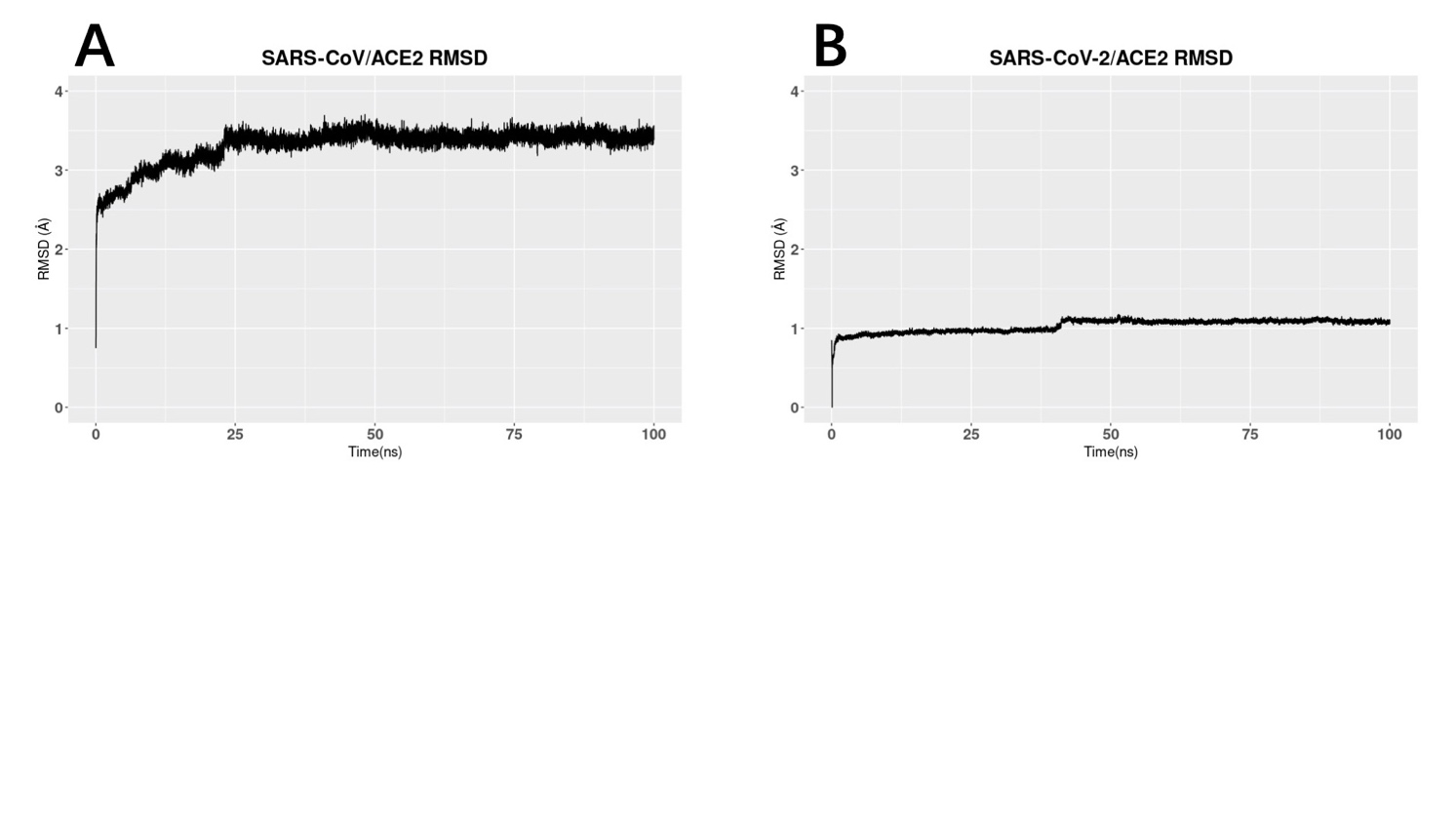


**Figure S1.** RMSD comparison of SARS-CoV/ACE2 and SARS-CoV-2/ACE2 complex structure

**
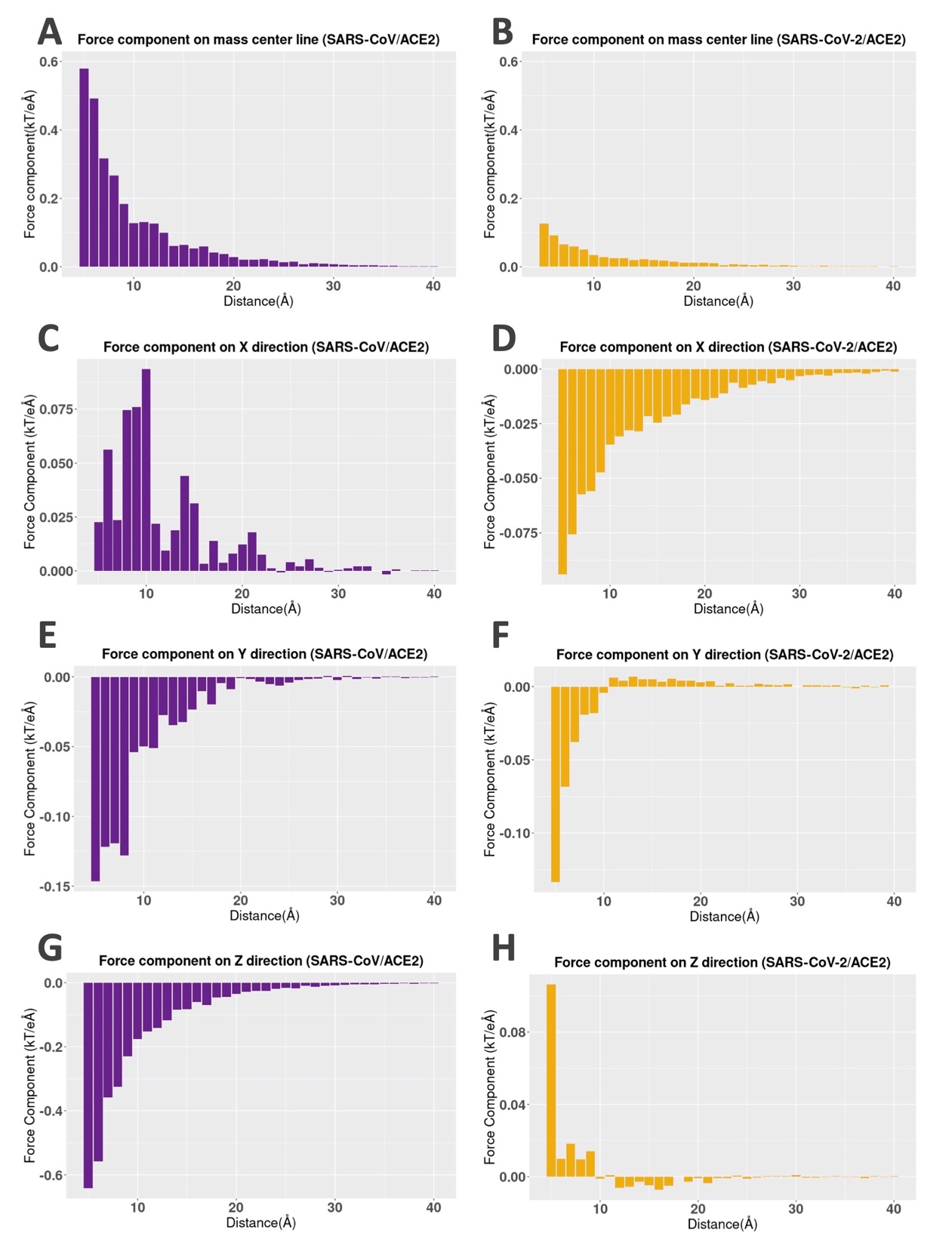
**

**Figure S2.** (A-B) The binding forces of SARS-CoV/hACE2 and SARS-CoV-2/hACE2 complexes (C-H) X,Y,Z components of electrostatic binding forces. The mass center line was set as the X axis.

**Movies:**

Movie1: SARS-CoV electrostatic surface

Movie2: SARS-CoV-2 electrostatic surface

Movie3: hACE2 electrostatic surface

Movie4: SARS-CoV RBD / hACE2 complex simulation

Movie5: SARS-CoV-2 RBD / hACE2 complex simulation

**Software applied:**

Delphi:

http://compbio.clemson.edu/delphi

DelphiForce:

http://compbio.clemson.edu/delphi-force/

DelphiPKa:

http://compbio.clemson.edu/pka_webserver/

NAMD:

https://www.ks.uiuc.edu/Research/namd/

R Studio:

https://www.rstudio.com/

Chimera:

https://www.cgl.ucsf.edu/chimera/
